# Dietary patterns and risk of ischemic stroke: A two-sample Mendelian randomization study

**DOI:** 10.1097/MD.0000000000045182

**Published:** 2025-10-17

**Authors:** Jingya Guo, Zhi Xi, Ming Wang, Xiaopeng Yang

**Affiliations:** a Department of Neurology, Zhengzhou University Affiliated Zhengzhou Central Hospital, Zhengzhou, Henan, China; b Department of Neurology, Zhengzhou University Affiliated Second Hospital, Zhengzhou, Henan, China.

**Keywords:** causal association, dietary habits, ischemic stroke, Mendelian randomization

## Abstract

Diet and nutrition critically influence the development and outcomes of ischemic stroke (IS). However, observational studies often yield inconsistent findings due to confounding and measurement error. Mendelian randomization (MR) provides an alternative approach to strengthen causal inference. We conducted a 2-sample MR analysis to evaluate the causal associations between 22 dietary factors and IS risk. Genetic instruments for dietary exposures were derived from the UK Biobank genome-wide association study, and outcome data were obtained from the MEGASTROKE consortium. Inverse-variance weighted analysis served as the primary method, complemented by sensitivity analyses. Consumption of oily fish (β = –0.402, *P* = .022), cheese (β = –0.364, *P* = .001), dried fruit (β = –0.710, *P* = .0002), weekly red wine (β = –0.507, *P* = .024), and calcium supplements (β = –3.994, *P* = .015) was associated with reduced risk of IS. Conversely, dietary patterns characterized by high-sugar or high-protein intake showed suggestive associations with increased IS risk, although these did not remain significant after multiple-comparison correction. This 2-sample MR study provides evidence that specific dietary factors, including oily fish, cheese, dried fruit, red wine, and calcium, may reduce IS risk, while high-sugar and high-protein diets may confer increased risk. These findings underscore the importance of dietary management in stroke prevention and highlight the need for further studies to validate the role of potentially harmful dietary patterns.

## 1. Introduction

Ischemic stroke (IS) is characterized by a sudden cessation of blood flow to the brain, continues to be a major contributor to global morbidity and mortality.^[1]^ As a leading cause of disability-adjusted life years, IS places a significant strain on healthcare systems and diminishes the quality of life for millions of people.^[2]^ The development of IS is complex, resulting from the complex interplay of genetic predisposition, environmental influences, and lifestyle choices, with diet increasingly recognized as a key modifiable contributor.^[3]^ Dietary habits influence cardiovascular health, metabolic processes, and inflammatory pathways, all of which are implicated in the development and progression of IS.^[4]^ Understanding the causal relationships between specific dietary components and IS risk is essential for developing targeted prevention strategies and informing public health policies.^[5]^ However, establishing causality in observational studies is challenging due to confounding factors, reverse causation, and measurement errors inherent in self-reported dietary data.^[6]^ To overcome these methodological constraints, Mendelian randomization (MR) has evolved as a powerful analytical framework in genetic epidemiology, employing genetic variants as instrumental variables (IVs) to establish causal relationships while substantially reducing confounding effects.

Nutrition and dietary habits are crucial for preventing and managing a wide range of chronic conditions, including cardiovascular diseases, diabetes, and stroke.^[7,8]^ Specific dietary components, such as fatty acids, micronutrients, and macronutrient profiles, have been hypothesized to modulate stroke risk through mechanisms such as lipid metabolism, blood pressure regulation, and oxidative stress. For instance, Omega-3 fatty acids, abundant in oily fish, are believed to decrease inflammation and enhance vascular health, potentially lowering IS risk.^[9]^ Similarly, dietary patterns rich in fruits, vegetables, and certain micronutrients, such as calcium, may confer protective effects by stabilizing endothelial function and reducing prothrombotic tendencies.^[10]^ Conversely, diets high in processed foods, sugars, or red meat may exacerbate risk factors like hypertension, dyslipidemia, and insulin resistance, which are established precursors to IS.^[11]^ Despite these associations, observational studies often yield inconsistent findings, partly due to variability in dietary assessment methods and residual confounding from socioeconomic or lifestyle factors.

The complexity of dietary habits, which encompass a wide range of foods, nutrients, and eating patterns, necessitates a comprehensive approach to evaluate their impact on IS. Previous research has explored individual dietary components, such as sodium intake or alcohol consumption, but few studies have systematically assessed a broad spectrum of dietary habits in relation to IS. Moreover, traditional epidemiological studies struggle to disentangle correlation from causation, as dietary patterns are often correlated with other risk factors, such as obesity or physical inactivity.^[12–15]^ MR provides an approach by employing genetic variants, randomly assigned at conception, as proxies for dietary factors. These genetic variants are minimally impacted by environmental biases and reverse causation, enabling a more reliable estimation of causal associations. By utilizing genome-wide association study (GWAS) datasets, this approach facilitates causal inference between nutritional factors and disease risk across diverse populations, providing evidence that overcomes inherent limitations of conventional observational research.^[16,17]^

The advent of large-scale biobanks, such as the UK Biobank, has revolutionized the study of diet–disease relationships by providing extensive genetic and phenotypic data.^[18]^ The UK Biobank, comprising over 500,000 participants, includes detailed dietary assessments and genetic information, making it an ideal resource for identifying genetic variants associated with dietary habits.^[19]^ These variants can serve as IVs in MR analyses to evaluate their causal impact on IS risk.^[20]^ Recent GWAS efforts, such as those from the MEGASTROKE consortium, have further enhanced our understanding of the genetic architecture of IS, identifying numerous single nucleotide polymorphisms (SNPs) associated with stroke risk.^[21,22]^ By integrating these datasets, a 2-sample MR approach can be employed, where genetic associations with dietary exposures are derived from 1 dataset (e.g., UK Biobank) and associations with IS are obtained from another (e.g., MEGASTROKE), maximizing statistical power and generalizability.

This study focuses on 22 dietary habits, ranging from specific food groups (e.g., oily fish, cheese, dried fruit) to beverage consumption (e.g., red wine) and dietary supplements (e.g., calcium). These exposures were selected to capture a diverse array of dietary patterns and nutrients relevant to cardiovascular health. IS, the outcome, was characterized using GWAS data from the MEGASTROKE consortium, comprising 34,217 cases and 406,111 controls of European descent. The core analytical approach, inverse-variance weighting (IVW) MR, was supported by sensitivity analyses, such as MR-Egger, weighted median, and MR-PRESSO, to confirm resilience against pleiotropy and heterogeneity. The choice of a 2-sample MR framework allows for the efficient use of publicly available summary statistics, reducing the need for individual-level data while maintaining analytical rigor.

The motivation for this study stems from the need to provide robust evidence for dietary interventions in IS prevention. While observational studies have suggested protective effects of certain foods, such as oily fish or fruits, and potential risks associated with high-sugar or high-protein diets, causal inferences remain limited. Furthermore, the role of dietary supplements, such as calcium, in stroke prevention is underexplored, despite their widespread use.^[23,24]^ By applying a diet-wide MR approach, this study aims to systematically evaluate the causal effects of multiple dietary habits on IS risk, addressing gaps in the literature and providing a foundation for personalized nutrition strategies. The findings have the potential to inform clinical guidelines, public health recommendations, and future research into the mechanisms underlying diet–disease relationships.

Therefore, the aim of this study was to systematically evaluate the potential causal relationships between 22 dietary habits and the risk of IS using a 2-sample MR design. By integrating genetic data from the UK Biobank and outcome data from the MEGASTROKE consortium, we sought to overcome key limitations of traditional observational studies and provide complementary evidence on the role of dietary patterns in IS. This approach may help identify dietary factors that are causally related to stroke risk and guide future preventive strategies.

## 2. Methods

**Figure 1.**
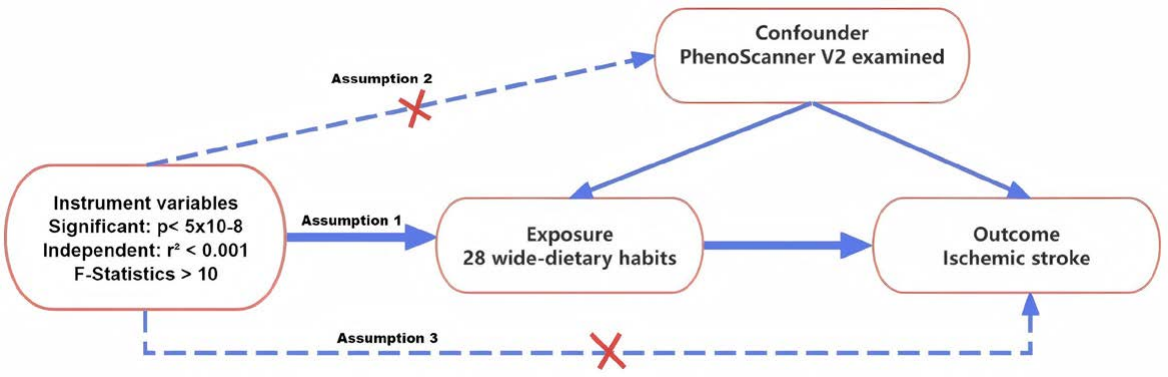
A directed acyclic graph is employed to depict the hypothesized influence of dietary habits on ischemic stroke. The use of a dotted line in this graph signifies the possibility of a direct causal relationship or a pleiotropic effect existing between the exposure and the outcome.

**Figure 2.**
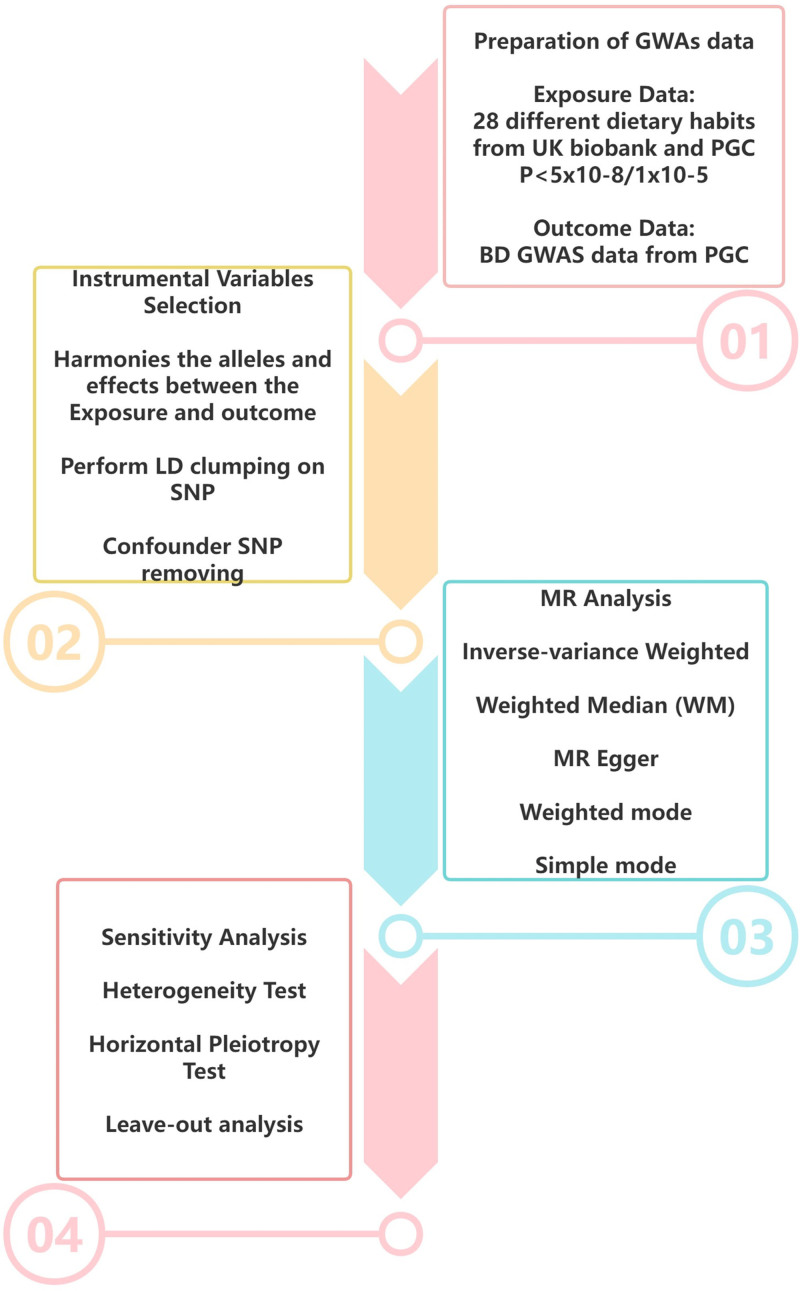
Summary of the experimental protocol.

This research examines the link between dietary patterns and IS. Figure 1 outlines the study design and data sources, while Figure 2 summarizes the experimental protocol employed. This investigation relies solely on aggregated GWAS summary statistics from open-access sources, thereby circumventing the need for further ethical review or participant consent procedures.

### 2.1. Exposure data

In this research, we examined 22 unique dietary patterns as exposure variables, selected thoughtfully based on prior literature. These exposures span 8 categories, encompassing processed meat, poultry, beef, pork, lamb/mutton, non-oily fish, oily fish, bread, dairy smoothie, cake, cheese, Snackpot, cereal, fresh fruit, dried fruit, cooked vegetables, raw salad vegetables, green tea, coffee, average weekly red wine, fizzy drinks, milk, pure fruit/vegetable juice, and mineral and other dietary supplements, including calcium.

The analysis utilized GWAS data from the UK Biobank, a population-based prospective cohort encompassing nearly 500,000 individuals aged 38 to 73 years, which offers comprehensive genetic and phenotypic characterization.^[25,26]^ The study methodology, including participant characteristics and quality control protocols, has been extensively described in prior publications.^[27,28]^ Both continuous and categorical variables were incorporated in the dietary pattern assessments, with implausible values systematically excluded during initial data processing. The GWAS summary statistics for dietary exposures were obtained from the MRC-IEU OpenGWAS database, a curated resource developed by Elsworth et al and maintained by the MRC Integrative Epidemiology Unit. Detailed information is available in Table 1.

**Table 1:** Detailed information on the GWAS datasets used in this MR study.

Exposure ID	Food types	Trait	Sample size	Consortium
ukb-b-6324	Meat	Processed meat intake	461,981	MRC-IEU (Elsworth et al)
ukb-b-8006		Poultry intake	461,900	
ukb-b-2862		Beef intake	461,053	
ukb-b-5640		Pork intake	460,162	
ukb-b-14179		Lamb mutton intake	460,006	
ukb-b-17627	Seafood	Non-oily fish intake	460,880	
ebi-a-GCST90096918		Oily fish consumption	460,443	
ukb-b-11348	Dairy and Sweet	Bread intake	452,236	
ukb-b-16139		Dark chocolate intake	64,945	
ukb-b-3433		Cake intake	64,949	
ukb-b-1489		Cheese intake	451,486	
ukb-b-12067		Sponge pudding intake	64,949	
ukb-b-15926	Grain	Cereal intake	441,640	
ukb-b-14690		White rice intake	64,949	
ukb-b-3881	Fruit	Fresh fruit intake	446,462	
ukb-b-16576		Dried fruit intake	421,764	
ukb-b-4070		Apple intake	64,949	
ukb-b-5362		Banana intake	64,949	
ukb-b-8887		Berry intake	64,949	
ukb-b-8089	Vegetable	Cooked vegetable intake	448,651	
ukb-b-1996		Salad/raw vegetable intake	435,435	
ukb-b-4078	Beverage	Green tea intake	64,949	
ukb-b-5237		Coffee intake	428,860	
ukb-b-5239		Average weekly red wine intake	327,026	
ukb-b-2832		Fizzy drink intake	64,949	
ukb-b-2966		Milk	64,943	
ukb-b-337		Purefruit/vegetable juice intake	64,949	
ukb-b-7043	Food additive	Mineral and other dietary supplements: calcium	461,384	
Outcome				
ID	Trait	Sample size	Consortium	
ebi-a-GCST006908	Ischemic stroke	440,328	MEGASTROKE	

GWAS = genome-wide association studies, MR = Mendelian randomization.

### 2.2. Outcome data

The IS outcome metrics were obtained from the MEGASTROKE consortium’s comprehensive GWAS meta-analysis, which aggregated data from multiple international studies to enhance statistical power and generalizability, accessed through the NHGRI-EBI GWAS Catalog. The MEGASTROKE dataset included 34,217 confirmed IS cases and 406,111 matched controls of European descent, with genome-wide analysis of 9.6 million SNPs. The IS phenotype was defined based on clinical diagnoses, typically adhering to the Trial of Org 10172 in Acute Stroke Treatment (TOAST) classification or equivalent diagnostic criteria, ensuring consistency in case ascertainment. The GWAS summary statistics provide genetic effect sizes (β coefficients), standard errors, and *P*-values for IS, enabling causal effect estimation in a 2-sample MR framework. To avoid bias due to heterogeneity across populations with different genetic backgrounds, this study exclusively utilized IS GWAS data related to European ancestry, ensuring consistency with the genetic background of the exposure data and thereby minimizing population stratification bias. The study utilized anonymized, open-access datasets that were exempt from additional ethics review. Rigorous quality control procedures were implemented, including the systematic removal of substandard SNPs to enhance analytical validity.

### 2.3. Selection of IVs

In this study, genetic instruments for dietary exposures were derived from UK Biobank datasets. The instrumental variable selection process adhered to the following criteria: first, genome-wide significant SNPs (*P* < 5 × 10⁻⁸) associated with each of the 22 dietary patterns were identified as potential instruments. Second, linkage disequilibrium (LD) pruning was performed using PLINK (*R*² threshold = 0.001, window size = 10,000 kb) with reference to European ancestry data from the 1000 Genomes Project.^[28]^ To address LD among SNPs, we kept the SNP with the lowest *P*-value. Third, to mitigate weak instrument bias, we computed *F*-statistics for all candidate instruments and excluded those with *F* < 10, applying the conventional threshold for instrument strength assessment.^[29]^ Following the alignment of exposure and outcome datasets and the removal of palindromic and weak instrumental variants, the remaining SNPs were utilized for MR analysis. When less than 3 exposure-associated SNPs were available, the *P*-value threshold for selection was lowered to 1 × 10^-5^. Additionally, PhenoScanner V2 was employed to assess relationships between chosen SNPs and possible confounding factors (*P* < 5 × 10^-5^).^[30]^ SNPs linked to confounders were removed from subsequent analyses.^[31]^

To further ensure instrument validity, all candidate SNPs were systematically screened for potential associations with established confounding traits using PhenoScanner V2. The confounders considered included body mass index, smoking, alcohol consumption, blood pressure, lipid levels, and diabetes-related traits, as these are known to influence both dietary habits and IS risk. SNPs showing significant associations (*P* < 5 × 10⁻⁵) with these confounders were excluded from subsequent analyses. IVs were required to satisfy 3 key MR assumptions: association strength: genetic variants must exhibit statistically significant associations with the exposure (*P* < 5 × 10^-8^) and sufficient instrumental strength (*F*-statistic > 10); independence: selected instruments must be independent of potential confounders (*r*² < 0.001 in LD clumping); exclusion restriction: genetic variants influence the outcome solely through the specified exposure.

To assess the relevance assumption, we quantified instrument strength by calculating both the variance explained (*R*²) and *F*-statistics for all genetic instruments. An *F*-statistic exceeding 10 was considered evidence of a robust IV.^[32]^ We utilized PhenoScanner V2 to remove SNPs linked (*P* < 5 × 10^-5^) to confounding factors, identified as traits unrelated to the exposure. Potential confounding variables are comprehensively documented in Table S1, Supplemental Digital Content, https://links.lww.com/MD/Q326. In summary, we evaluated and confirmed the appropriateness of IVs by computing *F*-statistics and *R*² values following the removal of SNPs linked to confounding factors. The exclusion restriction assumption is met when genetic instruments demonstrate no evidence of horizontal pleiotropy – the phenomenon whereby variants influence the outcome through pathways independent of the exposure. We assessed potential horizontal pleiotropy through MR-Egger regression analysis, where an intercept term statistically indistinguishable from zero (*P* > .05) suggested the absence of directional pleiotropic effects. We employed IVW, MR-Egger, weighted median, and weighted mode methods for analysis, with IVW as the primary approach, leveraging meta-analysis to combine individual SNP Wald ratios under the assumption that IVs influence the outcome solely through the specific exposure. This methodology operates under the assumption of balanced pleiotropy and demonstrates superior statistical efficiency among MR estimators. Heterogeneity in MR analysis, reflecting inconsistencies among IV estimates, was evaluated using Cochran *Q* test. Statistical evidence of heterogeneity (*P* < .05) prompted the adoption of a random-effects IVW model to account for between-SNP variability.

### 2.4. MR analysis

MR analyses were conducted using the 5 chief methods.^[32]^ The primary causal estimates were derived using the IVW method, with complementary sensitivity analyses performed through weighted median and MR-Egger regression approaches. The IVW approach assumes perfect instrument validity, implementing a fixed-effects meta-analysis of Wald ratios weighted by their inverse variances through a zero-intercept regression model. The final estimate represents the weighted average of all effect sizes. When the 3 core MR assumptions are met, IVW provides higher estimation precision and statistical power. In the absence of heterogeneity and pleiotropy, the IVW estimate is prioritized. The MR-Egger method differs from IVW by including an intercept term in the regression, enabling detection and adjustment for pleiotropic bias, while also using the inverse of the outcome variance as weights for fitting. In the presence of pleiotropy, MR-Egger results are prioritized. The weighted median method provides consistent causal estimates and can yield accurate results even when more than 50% of IVs are invalid. In cases of heterogeneity without pleiotropy, the weighted median estimate is prioritized. Heterogeneity across genetic instruments was formally assessed using Cochran *Q* statistic, with a significance threshold of *P* < .05 indicating substantial between-variant variability. When the *P*-value was < .05, a random-effects IVW model was selected as the final MR outcome; otherwise, a fixed-effects model was applied. Finally, we implemented the MR-PRESSO method to systematically detect and correct for horizontal pleiotropy, thereby validating the robustness of our IVW-derived causal estimates, removing palindromic SNPs with intermediate allele frequencies and outliers, performing a global test for heterogeneity and identifying horizontal pleiotropy.

### 2.5. Sensitivity analysis

Sensitivity analyses, including MR-Egger regression, MR-PRESSO, and leave-one-out tests, were conducted to verify the robustness of the IVW method. An MR-Egger intercept *P*-value < .05 indicated horizontal pleiotropy, prompting MR-PRESSO to detect and remove outliers, followed by reanalysis. Leave-one-out testing sequentially excluded each SNP to assess whether any single variant unduly affected the exposure–outcome relationship, ensuring result stability.

### 2.6. Statistical analysis

Our study examined 22 exposures across 8 dietary categories, a moderate scope for most MR studies. Additionally, we implemented a stringent Bonferroni correction to enhance the precision of our findings. Statistical significance was determined at *P* < .05 threshold, with results remaining significant after Bonferroni correction considered highly robust. All analytical procedures were performed using the TwoSampleMR package (version 0.5.6) in R statistical software (version 4.2.2, R Foundation for Statistical Computing, Vienna, Austria).

## 3. Results

Table 1 details each participating GWAS study. Our MR investigation evaluated causal relationships between nutritional factors and disease outcomes, utilizing genetic instruments that consistently demonstrated strong instrument strength (*F*-statistics > 10), thereby effectively minimizing potential weak instrument bias. Full MR results and sensitivity analyses appear in Table S2, Supplemental Digital Content, https://links.lww.com/MD/Q326. To evaluate the potential influence of individual variants, we performed leave-one-out analyses for each dietary exposure–outcome pair. The results showed that causal estimates were not substantially altered after excluding any single SNP, suggesting that the observed associations were not driven by a few influential data points.

### 3.1. Effect of dietary habits on IS

**Figure 3.**
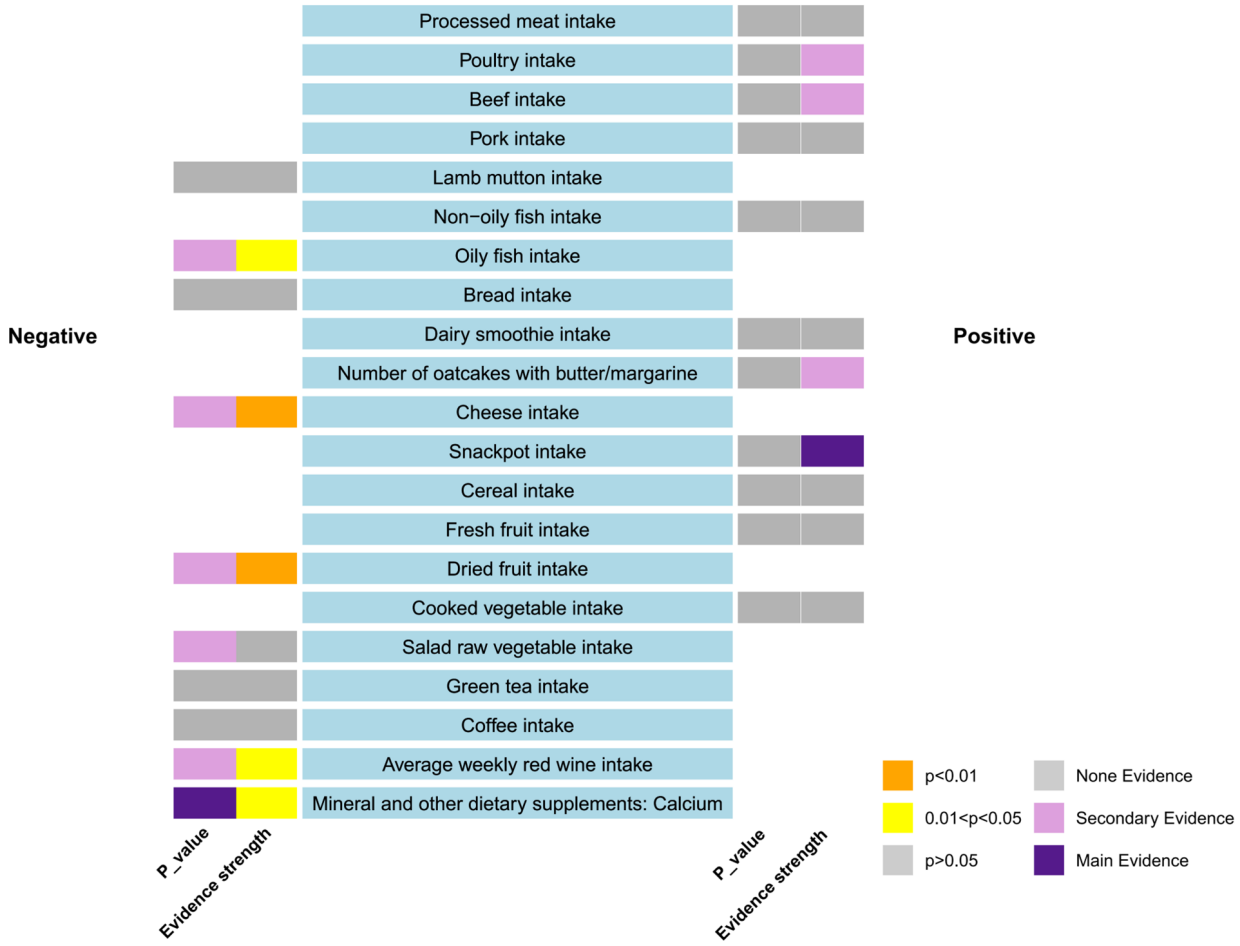
Summary results of association between dietary habits and ischemic stroke according to the IVW method. “Positive” represents the factors that have a risky effect on ischemic stroke, and “negative” represents the factors that have a protective effect on ischemic stroke, as defined by the beta value in the MR analysis.

**Figure 4.**
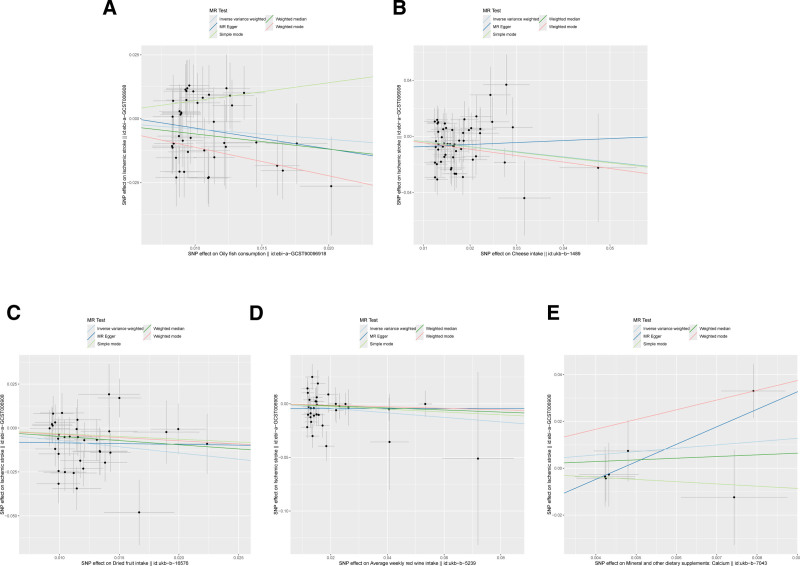
Scatter plots depicting the results of MR analyses investigating the association between dietary habits and ischemic stroke. Each line in the plot represents a different MR method, and the slope of each line represents the estimated association between the 2 variables. (A) Scatter plot between oily fish consumption and ischemic stroke; (B) scatter plot between cheese intake and ischemic stroke; (C) scatter plot between dried fruit intake and ischemic stroke; (D) scatter plot between average weekly red wine intake and ischemic stroke; (E) scatter plot between mineral and other dietary supplements: calcium and ischemic stroke.

The influence of dietary habits on IS is illustrated in Figure 3. Evidence levels were classified according to these results, with primary evidence demonstrating significant original *P*-values, while no evidence reflected a lack of significant associations. The strength of evidence for causal associations was interpreted according to statistical significance. Specifically, associations with *P*-values < .05 in the IVW analysis were considered to provide statistical evidence suggestive of a potential causal relationship, whereas associations with *P*-values ≥ .05 indicated no statistically significant evidence of association. This classification was applied only for descriptive purposes and should not be regarded as a formal grading of evidence. Based on these criteria, we identified several dietary patterns that showed significant associations with IS risk in the IVW analysis, while others demonstrated no statistically significant evidence of association. This systematic framework supports a prudent interpretation of the findings. Associations between 22 dietary habits and IS were evaluated using *P*-values from the inverse IVW method, as showed in Table 2. Our analysis revealed correlations between IS and 7 dietary habits: oily fish consumption (β = −0.402, SE = 0.176, *P* = .022), cheese intake (β = −0.364, SE = 0.113, *P* = .001), dried fruit intake (β = −0.710, SE = 0.189, *P* = .0002), average weekly red wine intake (β = −0.507, SE = 0.225, *P* = .024), and mineral and other dietary supplements: calcium (β = −3.994, SE = 1.637, *P* = .015). Scatter plots were generated to visualize the primary findings, the results are visually presented in Figure 4. In MR analysis, significant associations observed for single exposure–outcome pairs may indicate potential causal effects. However, given that 22 dietary exposures were tested, we applied Bonferroni correction (significance threshold: *P* < .0023) to reduce the likelihood of false-positive findings. Results that remained significant after correction were considered robust evidence (e.g., dried fruit intake), whereas associations that were nominally significant (*P* < .05 but not meeting the corrected threshold) should be interpreted as suggestive evidence only. This clarification ensures that our findings reflect both unadjusted and corrected statistical thresholds.

**Table 2:** MR results of the IVW method for the association of dietary habits with ischemic stroke.

Exposure	Number of SNPs	β	SE	*P*-value
Processed meat intake	21	0.011	0.172	.951
Poultry intake	7	0.549	0.441	.213
Beef intake	12	0.536	0.336	.111
Pork intake	11	0.054	0.375	.885
Lamb mutton intake	26	-0.053	0.217	.809
Non-oily fish intake	11	0.042	0.291	.884
Oily fish intake	42	-0.402	0.176	.022*
Bread intake	27	-0.116	0.163	.476
Dairy smoothie intake	3	0.268	0.376	.477
Number of oatcakes with butter/margarine	3	0.978	0.740	.187
Cheese intake	54	-0.384	0.114	.0007***
Snackpot intake	6	3.111	2.118	.142
Cereal intake	34	0.045	0.155	.772
Fresh fruit intake	49	0.126	0.221	.569
Dried fruit intake	35	-0.710	0.189	.0001***
Cooked vegetable intake	15	0.241	0.272	.376
Salad raw vegetable intake	11	-0.391	0.408	.337
Green tea intake	12	-0.003	0.005	.621
Coffee intake	35	-0.013	0.124	.918
Average weekly red wine intake	15	-0.507	0.225	.024*
Mineral and other dietary supplements: calcium	15	-3.994	1.637	.015*

IVW = inverse-variance weighting, MR = Mendelian randomization, SNPs = single nucleotide polymorphisms.

* *P* < .05.

*** *P* < .001.

**Figure 5.**
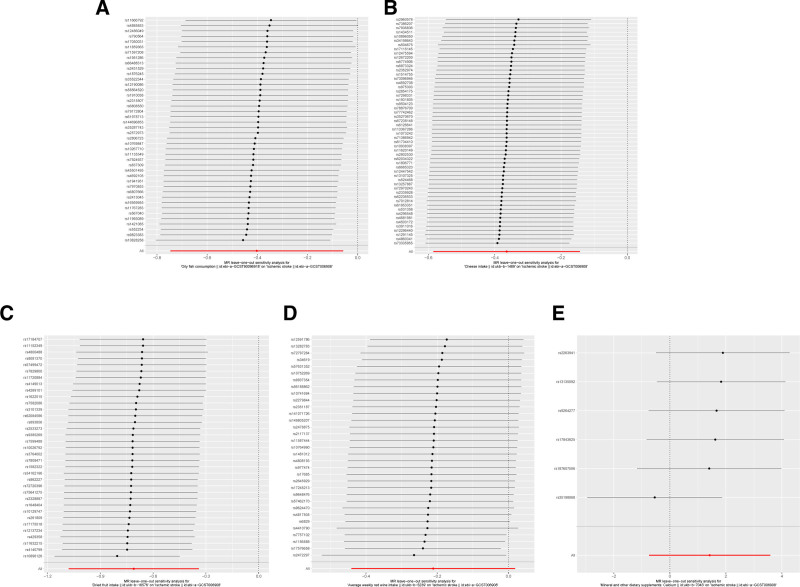
The results of a leave-one-out analysis on MR. Each black line in the figure corresponds to the outcome of the MR analysis when 1 SNP is removed from the analysis, while the remaining SNPs are used on the left. (A) Leave-one-out analysis between oily fish consumption and ischemic stroke; (B) leave-one-out analysis between cheese intake and ischemic stroke; (C) leave-one-out analysis between dried fruit intake and ischemic stroke; (D) leave-one-out analysis between average weekly red wine intake and ischemic stroke; (E) leave-one-out analysis between mineral and other dietary supplements: calcium and ischemic stroke.

**Figure 6.**
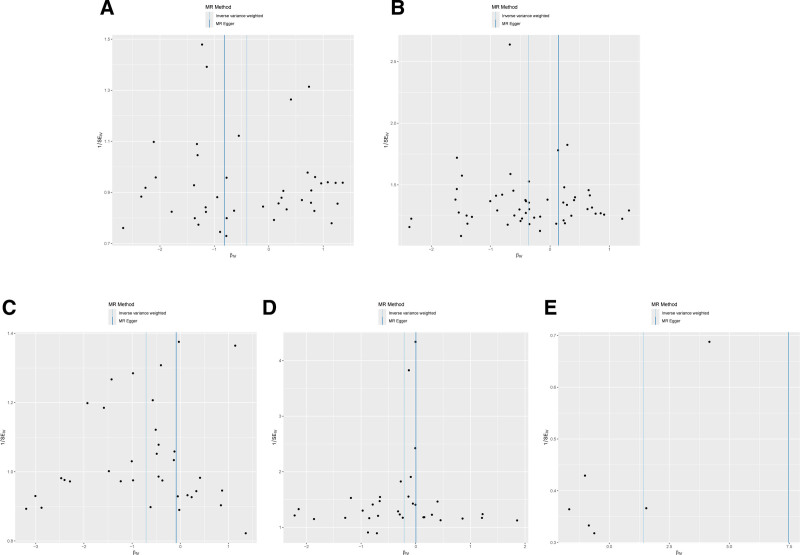
The results of funnel plots on MR. (A) Funnel plot between oily fish consumption and ischemic stroke; (B) funnel plot between cheese intake and ischemic stroke; (C) funnel plot between dried fruit intake and ischemic stroke; (D) funnel plot between average weekly red wine intake in food and ischemic stroke; (E) funnel plot between mineral and other dietary supplements: calcium and ischemic stroke.

**Figure 7.**
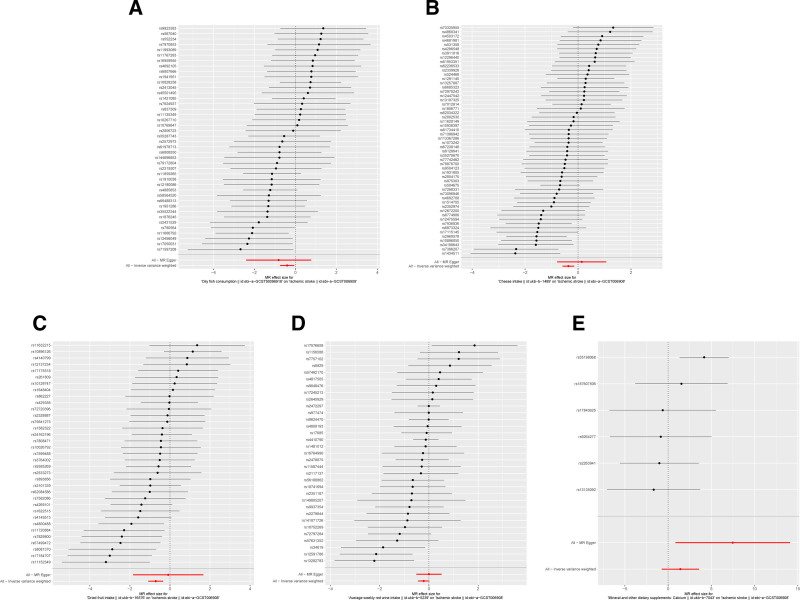
The results of forest plots on MR. (A) Forest plot between oily fish consumption and ischemic stroke; (B) forest plot between cheese intake and ischemic stroke; (C) forest plot between dried fruit intake and ischemic stroke; (D) forest plot between average weekly red wine intake in food and ischemic stroke; (E) forest plot between mineral and other dietary supplements: calcium and ischemic stroke.

Sensitivity analyses of the main findings included leave-one-out analysis. Exclusion of each significant SNP individually did not alter the statistical significance of the associations with IS, as depicted in Figure 5. The reliability of the IVW results was further confirmed by funnel plots and forest plots, provided in Figures 6 and 7.

## 4. Discussion

This study utilized a 2-sample MR framework to explore the causal relationships between 22 dietary habits and the risk of IS, drawing on GWAS data from the UK Biobank for dietary exposures and the MEGASTROKE consortium for IS outcomes. Our findings indicate that the consumption of oily fish, cheese, dried fruit, average weekly red wine, and calcium supplements may confer protective effects against IS, though the possibility of false positives requires cautious interpretation. Sensitivity analyses, including MR-Egger, weighted median, MR-PRESSO, and leave-one-out methods, supported the robustness of these results, suggesting minimal bias from pleiotropy or heterogeneity. These findings contribute valuable insights into the role of diet in IS prevention, highlighting potential dietary strategies while underscoring the need for further research to confirm and extend these observations.

The protective effect of oily fish consumption is consistent with the established cardiovascular benefits of omega-3 fatty acids, particularly eicosapentaenoic acid (EPA) and docosahexaenoic acid (DHA), which are prevalent in fish such as mackerel, sardines, and salmon.^[33]^ These fatty acids are known to modulate inflammatory pathways, enhance endothelial function, and reduce thrombotic tendencies, all of which are critical in preventing IS.^[34]^ Observational studies, such as those from the Cardiovascular Health Study, have reported associations between higher fish intake and lower stroke risk, but these studies are often limited by confounding factors, such as lifestyle or dietary patterns.^[35]^ By using genetic variants as proxies for oily fish consumption, our MR analysis mitigates these confounders, providing stronger evidence for a causal protective effect. This finding supports dietary recommendations to increase oily fish intake, particularly for individuals at elevated risk of stroke due to hypertension or dyslipidemia.^[36]^ However, the optimal frequency and quantity of fish consumption remain to be clarified, as excessive intake may introduce concerns about mercury exposure or sustainability.

The potential protective role of cheese consumption is a novel finding that warrants further exploration. Cheese is a complex food, containing calcium, vitamin K2, and saturated fats, which may have competing effects on vascular health.^[37]^ Calcium and vitamin K2, particularly in aged or fermented cheeses, may reduce arterial stiffness and inhibit vascular calcification, a key contributor to IS. Conversely, the high saturated fat content in cheese has been linked to adverse lipid profiles in some studies, raising questions about its overall cardiovascular impact. Our MR results suggest that the beneficial components of cheese may outweigh its potential risks in the context of IS, possibly due to its micronutrient profile. This finding challenges some observational studies that associate dairy consumption with increased cardiovascular risk, highlighting the advantage of MR in isolating causal effects. Future research should investigate specific cheese types and their bioactive components to better understand this association and inform dietary guidelines.

Dried fruit consumption, identified as potentially protective, aligns with the broader evidence supporting fruit intake for cardiovascular health.^[38]^ Dried fruits, such as raisins, apricots, and prunes, are rich in dietary fiber, antioxidants, and potassium, which may reduce IS risk by improving lipid metabolism, reducing oxidative stress, and stabilizing blood pressure. Observational studies, including those from the Health Professionals Follow-up Study, have linked higher fruit consumption to lower stroke incidence, but self-reported dietary data are prone to measurement errors.^[39,40]^ Our MR approach, using genetic instruments from the UK Biobank, strengthens the causal inference by minimizing such biases. The concentrated nutrient profile of dried fruits makes them a practical option for dietary interventions, particularly in populations with limited access to fresh produce. However, the high-sugar content in some dried fruits raises concerns about glycemic load, necessitating studies to determine optimal portion sizes and types of dried fruit for stroke prevention.

The association between moderate red wine consumption and reduced IS risk resonates with the cardiovascular benefits attributed to moderate alcohol intake, often termed the “French paradox.” Red wine contains resveratrol, flavonoids, and other polyphenols that exhibit antioxidant, anti-inflammatory, and vasodilatory properties, potentially protecting against cerebral ischemia. Observational studies have reported a J-shaped relationship between alcohol consumption and stroke risk, with moderate intake linked to lower risk and heavy drinking associated with increased risk.^[41,42]^ Our MR analysis, by using genetic variants associated with red wine consumption, provides evidence of a causal protective effect at moderate levels, mitigating biases from drinking patterns or socioeconomic factors. However, the potential for false positives, as noted in our results, underscores the need for replication studies to confirm this effect. Public health messaging must balance these findings with warnings against excessive alcohol consumption, which is a known risk factor for hemorrhagic stroke and other adverse outcomes.^[43]^

The protective effect of calcium supplements is a significant finding with implications for both clinical practice and public health. Calcium plays a critical role in vascular homeostasis, influencing blood pressure regulation and endothelial function. Observational studies have produced conflicting results, with some suggesting that calcium supplements may increase cardiovascular risk due to vascular calcification, particularly in older adults.^[44,45]^ Our MR results support a protective role for calcium supplements in IS prevention, potentially mediated through their effects on hypertension or vascular stability. The use of genetic instruments minimizes confounding by health-seeking behaviors, which often complicate observational studies of supplement use. However, the possibility of pleiotropic effects, where genetic variants for calcium supplement intake influence other traits, cannot be fully excluded. Sensitivity analyses, including MR-PRESSO, helped address this concern, but further studies are needed to clarify the mechanisms and optimal dosing of calcium supplementation for stroke prevention.

The methodological strengths of this study enhance the reliability of our findings. The 2-sample MR framework, utilizing large-scale GWAS data from the UK Biobank and MEGASTROKE consortium, provided substantial statistical power to detect causal effects.^[46]^ The primary IVW method was complemented by robust sensitivity analyses, including MR-Egger, weighted median, and MR-PRESSO, which confirmed the absence of significant pleiotropy or heterogeneity in most analyses.^[47]^ The restriction to European ancestry populations ensured genetic homogeneity between exposure and outcome datasets, reducing population stratification bias. Additionally, the use of publicly available, de-identified data eliminated the need for additional ethical approvals, facilitating efficient and ethical research. The comprehensive evaluation of 28 dietary habits allowed for a broad assessment of diet’s role in IS, addressing a gap in the literature where most studies focus on single nutrients or foods.

Several limitations of this study should be noted. First, although MR reduces certain biases such as reverse causation and unmeasured confounding, it does not completely eliminate them. The associations between genetic variants and dietary habits that serve as IVs remain correlational, and thus may not fully capture the complexity of dietary behaviors. Moreover, dietary assessments in the UK Biobank are subject to measurement heterogeneity, which may introduce bias similar to that encountered in traditional observational studies. Socioeconomic and lifestyle factors may also influence both dietary choices and IS risk, and although we employed statistical adjustments and sensitivity analyses (e.g., MR-Egger, MR-PRESSO), residual confounding cannot be entirely excluded. Therefore, MR should be regarded as a complementary approach that provides supportive evidence rather than definitive causal proof.

Second, the dietary categories analyzed were relatively broad. Variables such as “cheese” or “fruit” represent heterogeneous groups that encompass multiple food subtypes with distinct nutritional profiles and biological effects. For instance, fresh fruit and dried fruit, or different types of cheese, vary considerably in sugar, fat, micronutrient, and bioactive compound composition, which could influence IS risk in different ways. Due to the structure of the UK Biobank dietary questionnaires and the available GWAS summary statistics, we were unable to stratify these categories further. Future studies incorporating more granular dietary assessments or biomarker-based measures are warranted to provide more specific and clinically relevant insights.

Taken together, these limitations suggest that our findings should be interpreted with caution. Further research in diverse populations, with refined dietary categorizations and complementary study designs, will be essential to confirm and extend the present results.^[48]^

The implications of our findings are 2-fold, they inform dietary recommendations for IS prevention and highlight the utility of MR in nutritional epidemiology.^[49]^ The protective effects of oily fish, cheese, dried fruit, red wine, and calcium supplements suggest that targeted dietary interventions could reduce IS incidence, particularly in high-risk populations. For instance, incorporating oily fish and dried fruit into dietary guidelines could be a cost-effective strategy, while moderate red wine consumption may be cautiously recommended for individuals without alcohol-related contraindications. Calcium supplementation, however, requires careful consideration due to potential risks in certain groups, such as those with preexisting cardiovascular disease. Clinicians should integrate these findings into personalized nutrition plans, emphasizing balance and moderation. From a research perspective, our study demonstrates the power of MR to overcome the limitations of observational studies, providing a model for future diet–disease investigations.

Future research should prioritize several directions. First, replication studies using independent GWAS datasets or novel genetic instruments could validate our findings and address the risk of false positives. Second, MR analyses in diverse populations, including Asian, African, and Hispanic cohorts, are essential to assess the generalizability of our results. Third, mechanistic studies exploring the pathways linking specific dietary components to IS risk could inform targeted interventions and clarify dose-response relationships. Fourth, multivariable MR approaches could disentangle the effects of correlated dietary habits or mediators, such as body mass index or lipid profiles, particularly for high-sugar and high-protein diets, which our study identified as areas needing further exploration. Finally, integrating MR with longitudinal cohort studies could provide a more comprehensive understanding of how dietary patterns evolve over time and influence IS risk.

In conclusion, this study provides evidence of causal associations between specific dietary habits (oily fish, cheese, dried fruit, red wine, and calcium supplements) and reduced IS risk, using a robust 2-sample MR framework. These findings highlight the potential of dietary interventions to prevent IS, while the robustness of our sensitivity analyses strengthens confidence in the results. However, the possibility of false positives and limitations in generalizability underscore the need for cautious interpretation and further research. By addressing these gaps, future studies can refine dietary recommendations and advance our understanding of diet’s role in stroke prevention, particularly for complex dietary patterns like high-sugar and high-protein diets. This work underscores the importance of integrating genetic epidemiology with nutritional science to inform evidence-based strategies for reducing the global burden of IS.

## 5. Conclusions

This MR study provides evidence supporting potential causal relationships between specific dietary habits, namely oily fish, cheese, dried fruit, red wine, and calcium supplementation and reduced risk of IS. By leveraging genetic instruments and large-scale GWAS datasets, the analysis reduces confounding and reverse causation inherent in observational studies. These findings underscore the importance of diet in stroke prevention and may inform future clinical guidelines and public health strategies. Further research in diverse populations and across varying dietary exposures is essential to validate these associations and clarify underlying biological mechanisms.

## Acknowledgments

We extend our gratitude to all participants and researchers involved in this MR study. Additionally, we acknowledge the Genome-Wide Association Studies (GWAS) databases for pro viding publicly available summary data.

## Author contributions

**Conceptualization:** Xiaopeng Yang.

**Data curation:** Jingya Guo, Zhi Xi, Ming Wang.

**Formal analysis:** Jingya Guo, Zhi Xi.

**Investigation:** Ming Wang.

**Methodology:** Jingya Guo, Zhi Xi, Ming Wang, Xiaopeng Yang.

**Project administration:** Xiaopeng Yang.

**Resources:** Xiaopeng Yang.

**Software:** Zhi Xi, Ming Wang.

**Visualization:** Jingya Guo, Zhi Xi, Ming Wang, Xiaopeng Yang.

**Writing – original draft:** Jingya Guo.

**Writing – review & editing:** Jingya Guo, Zhi Xi, Ming Wang, Xiaopeng Yang.

## Supplementary Material


